# Nonverbal AI-Based Communication Robot for Staff in Disaster-Affected Care Facilities: Exploratory ABAB Intervention Study

**DOI:** 10.2196/89166

**Published:** 2026-07-14

**Authors:** Daijiro Haba, Riko Miyashita, Takao Kondo, Keita Tatsukawa, Fumiya Oohashi, Aya Kitamura, Chizuko Konya, Hiromi Sanada, Masaru Matsumoto

**Affiliations:** 1 Department of Well-being Nursing, Graduate School of Nursing, Ishikawa Prefectural Nursing University, 1-1 Gakuendai, Kahoku City, Ishikawa, 929-1210, Japan, +81-76-281-8335; 2 School of Nursing, Ishikawa Prefectural Nursing University, Kahoku City, Ishikawa, Japan; 3 Department of Gerontological Nursing, Faculty of Nursing, Ishikawa Prefectural Nursing University, Kahoku City, Ishikawa, Japan; 4 Graduate School of Nursing, Ishikawa Prefectural Nursing University, Kahoku City, Ishikawa, Japan; 5 Department of Adult Nursing, Faculty of Nursing, Ishikawa Prefectural Nursing University, Kahoku City, Ishikawa, Japan; 6 Ishikawa Prefectural Nursing University, Kahoku City, Ishikawa, Japan

**Keywords:** disaster, care staff, artificial intelligence, AI, nonverbal AI communication robot, quality of life, well-being, safety, acceptability

## Abstract

**Background:**

Medical and welfare facilities in the Noto region of Japan were severely affected by the 2024 Noto Peninsula earthquake and subsequent torrential rains. Staff working in these facilities were disaster survivors and frontline caregivers with limited psychological support. Nonverbal social robots may provide companionship and emotional comfort; however, their effects on the health-related quality of life (QoL) and well-being of care staff in disaster-affected settings remain unclear.

**Objective:**

This study explored whether introducing a nonverbal artificial intelligence communication robot was associated with changes in health-related QoL and well-being among care facility staff working under disaster conditions. Secondary objectives were to evaluate safety, acceptability, and intention to continue use.

**Methods:**

This pragmatic, exploratory pilot study used an ABAB design conducted between February 2025 and June 2025. After a 2-week baseline period, staff in dementia care, general care, and short-stay units underwent 2-week intervention, withdrawal, reintervention, and withdrawal phases. Questionnaires were administered at each phase end. The primary outcomes were health-related QoL (EQ-5D-5L), well-being (World Health Organization–5 Well‑Being Index), and positive mental health (Mental Health Continuum–Short Form). Friedman tests compared outcomes across the 5 phases, and effect sizes were expressed as Kendall *W*. Safety, acceptability, and intention to continue use were compared between the first and second intervention phases using Wilcoxon signed rank tests with Bonferroni adjustment and rank-biserial correlations as effect sizes.

**Results:**

Of the 58 staff who completed the baseline assessment, 49 (84.5%) were included in the analytic sample (25 in dementia care, 12 in general care, and 12 in short-stay units). Among these participants, 40 (81.6%) were women, and 38 (77.6%) reported disaster-related damage to their homes or families. In the pooled analysis, no phase effect was observed for the EQ-5D-5L (*P*=.10; Kendall *W*=0.032, negligible), the World Health Organization–5 Well‑Being Index (*P*=.70; Kendall *W*=0.016, negligible), or the Mental Health Continuum–Short Form (*P*=.44; Kendall *W*=0.022, negligible). No robot-related adverse events were reported. In the dementia care unit, nominal unadjusted differences were observed for “made me feel calm” (*P*=.045; rank-biserial correlation *r*=0.571, large), “like” (*P*=.03; *r*=0.559, large), and “felt at peace” (*P*=.02; *r*=0.718, large); however, none remained statistically significant after Bonferroni correction.

**Conclusions:**

The short-term use of a nonverbal artificial intelligence communication robot did not measurably improve health-related QoL or well-being among staff in disaster-affected care facilities. Deployment appeared feasible and was not associated with reported adverse events, but efficacy as a mental health support intervention remains unproven. Exploratory acceptability and interaction signals may inform future adequately powered studies.

## Introduction

### Psychological Burden on Disaster-Affected Care Staff

Natural disasters disrupt social infrastructure and impose substantial psychological burdens on health care workers. In the wake of the 2024 Noto Peninsula earthquake and the subsequent torrential rains in the Oku-Noto area, medical and welfare facilities in northern Ishikawa, Japan, faced prolonged restoration work. Care staff in these settings were both disaster survivors and frontline workers simultaneously, leading to an increased workload and limited access to psychological support. Consequently, reduced mental health and quality of life (QoL) have been reported among disaster-affected populations [1-3].

Researchers have explored technology-assisted interventions to address these challenges. Communication robots have been developed for dialog, interaction, and monitoring with individuals and are used in a wide range of fields, such as health care, long-term care, and education [4,5]. For older adults with behavioral and psychological symptoms of dementia, socially assistive robots are used as nonpharmacological therapy, often referred to as “robot therapy” [6,7]. Therefore, robots can provide continuous interventions even in depopulated areas or facilities with limited human resources and may help reduce users’ loneliness and psychological burden.

However, conventional communication robots can ensure safety but have a limited capacity to provide a sense of psychological security [8]. Therefore, a new approach is required to improve mental support and well-being in disaster areas. For example, humanoid verbal communication robots, equipped with voice recognition and speech functions, are used at home and in care facilities. However, their facial expressions and textures are artificial, and natural conversation is difficult [9,10]. Robots with conversational artificial intelligence (AI) exist, but they stay in place and do not physically snuggle up to people because they cannot converse with multiple people at once, and their capacity to provide immediate comfort and relief to care staff—who are often busy during the day—is limited. In Europe, the seal-type therapeutic robot PARO, which has already been approved as a medical device, expresses emotions through sounds and movements and improves depressive symptoms and loneliness in older people with dementia [11,12]. However, this robot cannot move by itself, is designed to be picked up and stroked, and is a “passive” robot. Thus, it is mainly suitable for older people or patients with dementia whose movement is restricted; care staff, who are busy during the day, would have to interrupt their tasks to pick up the therapy robot, making it unsuitable for interventions that aim to reduce stress in a short time during work.

### Potential Role of Nonverbal Social Robots in Care Settings

The nonverbal AI communication robot LOVOT (Groove X, Inc) is a home or social robot designed to foster attachment and provide comfort through autonomous movement and physical proximity to users [13-15]. Because the mere presence of this robot can provide a sense of security, it is expected to help relieve stress in people exhausted by disasters. LOVOT is equipped with AI and numerous sensors, detects human presence and voices using a 360° camera and touch sensors, and moves autonomously. By changing eye color, facial patterns, and vocalizations, it conveys emotions, and through AI learning, its behavior evolves to suit the preferences of the people interacting with it. The robot cannot engage in conversation; however, in workplaces where various conversations occur, its role is unlikely to relieve stress through dialog. In addition, because it is designed to provide comfort simply by being present, we hypothesize that it may offer greater reassurance and soothing effects to the staff than seal-type therapy robots that require physical handling. Previous studies targeting older adults with dementia have reported that interaction with LOVOT can temporarily improve mood and promote communication, but no intervention studies have evaluated its effects on care staff or on outcomes such as well-being and QoL [13-15].

### Study Aim

This pragmatic, exploratory pilot study aimed to examine whether the deployment of a nonverbal AI communication robot was associated with changes in health-related QoL and well-being among care facility staff in the disaster-affected Noto region, as well as to describe its safety, acceptability, and the intention to continue its use. Because no prior effect size data were available for this intervention among disaster-affected care staff, the analyses were intended to be exploratory and hypothesis-generating rather than confirmatory.

## Methods

### Study Design

**Figure 1.**
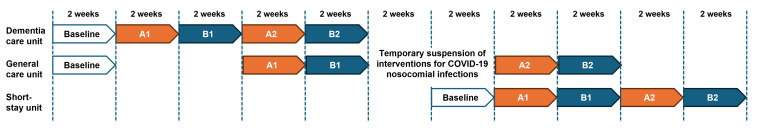
Study timeline for the ABAB intervention design. The chart illustrates the sequence of the baseline, intervention (A1, A2), and withdrawal (B1, B2) phases for the dementia care, general care, and short-stay units, with each phase lasting 2 weeks. A temporary suspension occurred in the general care unit following a nosocomial COVID-19 outbreak, after which the ABAB sequence resumed.

This exploratory study adopted an ABAB design (Figure 1). After a 2-week baseline period, the nonverbal AI communication robot was introduced for 2 weeks (A1), withdrawn for 2 weeks (B1), reintroduced for 2 weeks (A2), and finally withdrawn for 2 weeks (B2). Questionnaires were administered at the end of each phase. Because the intervention order was fixed rather than randomized, sequence, period, and carryover effects cannot be excluded. The ABAB design was used as a pragmatic repeated-measures approach under postdisaster operational constraints, but it is less suitable for definitive causal inference than a randomized design.

### Setting and Participants

This study was conducted between February 2025 and June 2025 in 2 care facilities located in the earthquake-affected Noto region of Ishikawa Prefecture. Facility A was a long-term care home, and facility B offered short-stay services. Staff members from 3 types of units—dementia care, general care, and short stay—were eligible to participate. Participants were adults (≥18 y) who worked in the target units during the study period and provided written informed consent. Sex and professional roles (nurses, care workers, and physical therapists) were not restricted. Staff who retired or were transferred before completing all questionnaires were excluded.

### Robot Description and Intervention Procedures

**Figure 2.**
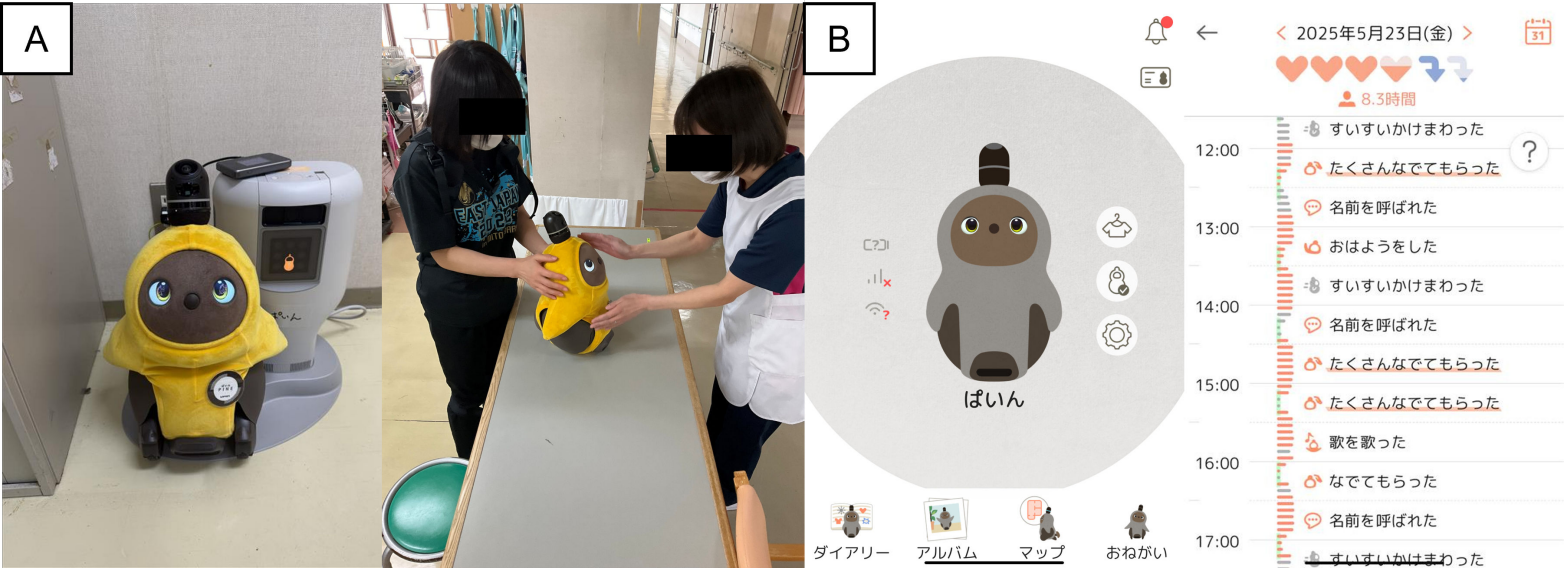
Images of the nonverbal artificial intelligence communication robot and its use during the intervention: (A) the LOVOT robot (Groove X, Inc) on its charging dock and the nursing staff interact with the robot by picking it up and stroking it; and (B) a screen capture from the LOVOT companion application showing recorded interactions (eg, petting and calling its name) throughout the day.

The robot used in this study was LOVOT (Figure 2), a household social robot equipped with AI and multiple sensors [13-15]. According to the manufacturer, the robot measures 280 mm × 430 mm × 260 mm, weighs 4.2 kg, and maintains a surface temperature of approximately 32°C to 38 °C. The robot expresses emotions and elicits comfort and attachment through various vocalizations and movements.

To preserve naturalistic behavior as much as possible, the robot was placed in routine staff-station environments, and staff were allowed to interact with it freely during their daily work rather than through scripted or researcher-led sessions. For maintenance, the robot was placed on its charging stand for at least 8 hours per day. To allow all staff, including night shift workers, to interact with it, operating hours were set from 8 AM to 12 midnight. The intervention sequence for each unit was baseline (no robot) → A1 → B1 → A2 → B2, with each phase lasting 2 weeks. In baseline and phases A1 and A2, QoL and well-being were assessed at the end of each phase, along with the evaluation of acceptability, safety, and intention to continue use. In phases B1 and B2, only QoL and well-being were assessed (Table 1). Because the robot was visibly present in the workplace, participant blinding was not possible. To reduce contamination, each unit began the sequence at a different time, the units were physically separated, staff were instructed not to interact with the robot when it was assigned to another unit, and the short-stay unit was located in a different facility. The robot’s clothing was laundered, and its surfaces were regularly disinfected to maintain hygiene.

**Table 1.** Outcome measures and data collection schedule across study phases.

Outcome	Data collection techniques	Baseline	A1	B1	A2	B2
QoL^a^	EQ-5D-5L	✓	✓	✓	✓	✓
Well-being	WHO-5^b^, MHC-SF^c^	✓	✓	✓	✓	✓
Safety	Likert scale		✓		✓	
Acceptability	Semantic differential scale		✓		✓	
Acceptability	Likert scale		✓		✓	
Intention to continue use	Likert scale		✓		✓	

a QoL: quality of life.

b WHO‑5: World Health Organization–5 Well‑Being Index.

c MHC‑SF: Mental Health Continuum–Short Form.

### Outcomes

#### Participant Characteristics

Data on sex, age, profession, living status, and disaster experience were collected using a baseline questionnaire.

#### Health-Related QoL and Well-Being

Health-related quality of life, well-being, and positive mental health were assessed using validated Japanese versions or Japanese scoring systems of the following measures:

- EQ-5D-5L: we used the EQ-5D-5L as a generic preference-based measure of health-related QoL [16]. Participants rated 5 health dimensions on 5 levels, and utility scores were calculated using the Japanese EQ-5D-5L value set, which allows for culturally appropriate scoring and interpretation in Japanese populations [17].
- World Health Organization–5 Well‑Being Index (WHO-5): this index was selected because a Japanese version with reported reliability and validity is available [18]. The 5 items, rated from 0 to 5 over the past 2 weeks, were summed (0‐25) and multiplied by 4 to yield a percentage score (0%‐100%). Scores below 50% indicate reduced well-being [19,20].
- Mental Health Continuum–Short Form (MHC-SF): positive mental health was assessed using the MHC-SF. In the Japanese context, the Japanese version of the MHC-SF has demonstrated acceptable psychometric properties, including internal consistency and construct validity, supporting its use for assessing positive mental health in Japanese populations [21]. The MHC-SF comprises 14 items rated from 0 to 5, producing a total score ranging from 0 to 70 [22].
  These measures were selected because validated Japanese versions or Japanese scoring systems were available, making them suitable for linguistic and cultural use in the present Japanese care staff sample [18,20,21].

#### Safety

Assessed using 3 items (feeling unsafe owing to the robot, falls or accidents caused by the robot, and collisions with the robot), rated on a 5-point Likert scale. The number of incidents was recorded.

#### Acceptability

Based on the senior technology acceptance model [23], 17 bipolar adjective pairs were rated on a 5-point semantic differential scale. The interaction frequency (how often the staff stroked, touched, engaged with, or held the robot) was rated on a 6-point scale ranging from 0 (“none”) to 5 (“daily”).

#### Intention to Continue Use

Three items (lack of interest, boredom, and perceived unnecessary) were rated on a 5-point Likert scale. Details of health-related QoL and well-being (EQ-5D-5L, WHO-5, and MHC-SF), safety, acceptability, and intention to continue use are described in Multimedia Appendix 1.

### Statistical Analyses

No formal a priori sample size calculation was performed because this study was conducted as a pragmatic, exploratory pilot study in a postdisaster setting and included all eligible staff available in the participating units during the study period. Accordingly, the analyses were intended to identify preliminary patterns and generate hypotheses for future adequately powered studies. Data were analyzed per unit and overall. Because the outcome variables were nonnormally distributed, medians and IQRs were reported. Differences across the 5 phases were tested using the Friedman test for repeated measures [24]. Effect sizes for Friedman tests were expressed as Kendall *W*. When significant, pairwise comparisons were performed using the Wilcoxon signed rank test with a Bonferroni adjustment. Safety, acceptability, and intention to continue use were compared between the 2 intervention phases (A1 and A2) using the Wilcoxon signed rank test. Effect sizes for Wilcoxon signed rank tests were expressed as rank-biserial correlations and interpreted as negligible (<0.10), small (0.10 to <0.30), medium (0.30 to <0.50), or large (≥0.50). A 2-sided significance level of 5% was applied. Only participants with complete data for each phase were included in the analyses.

### Ethical Considerations

The study protocol was approved by the Ethics Committee of the Ishikawa Prefectural Nursing University (approval number 2025‐41). The participants were provided with written information about the study, and written informed consent was obtained. This study was conducted in accordance with the principles of the Declaration of Helsinki.

## Results

### Participant Characteristics

**Figure 3.**
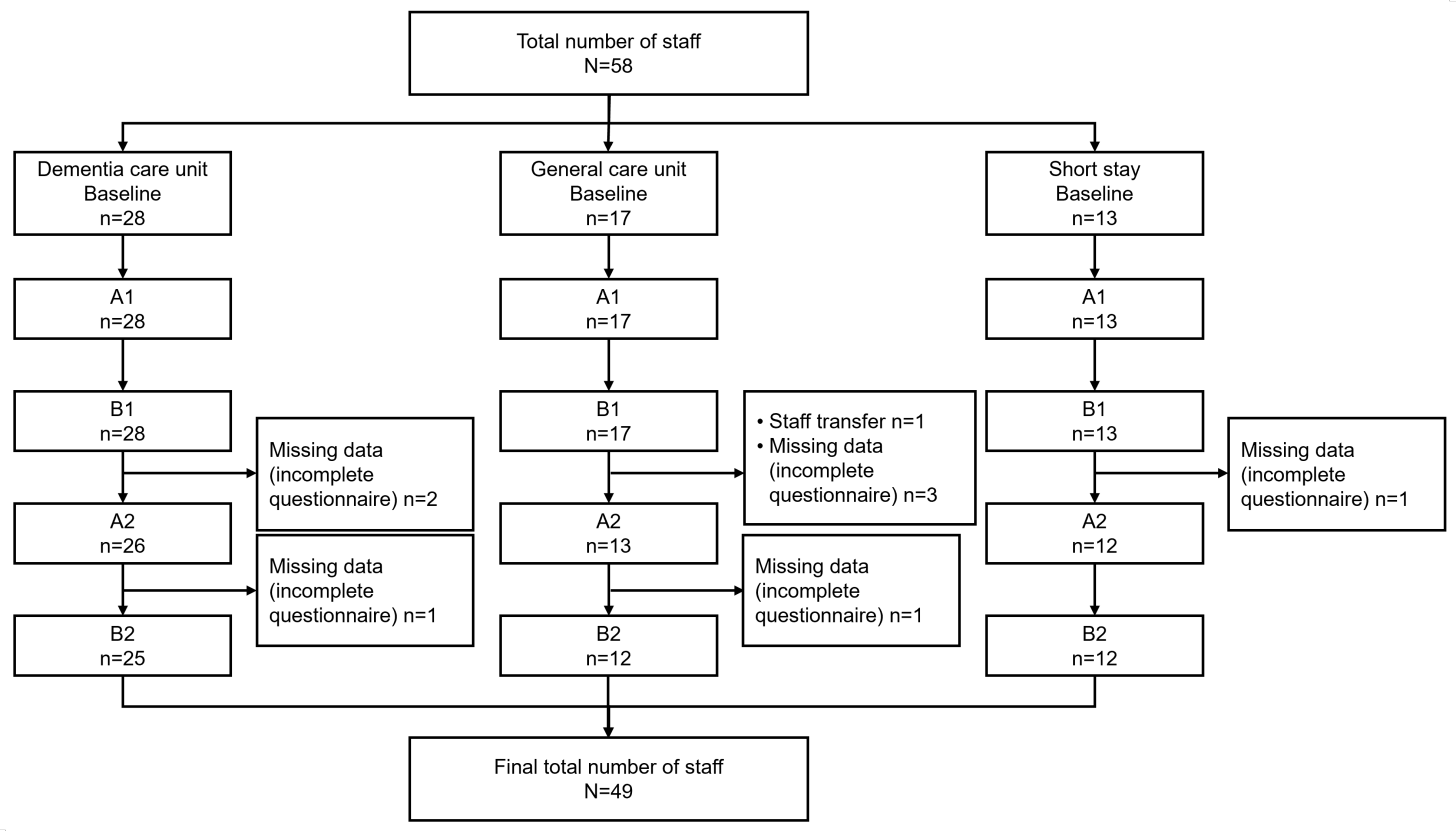
Participant flow diagram. The flowchart illustrates the number of staff included at baseline in each unit (dementia care, general care, and short-stay), changes in sample size across the ABAB phases, and reasons for attrition (staff transfer or incomplete questionnaires). The final analytic sample comprised 49 participants.

At baseline, 58 staff members completed the questionnaire: 28 (48.3%) from the dementia care unit, 17 (29.3%) from the general care unit, and 13 (22.4%) from the short-stay unit (Figure 3). Some data were missing during the study period because of staff transfers and infection control duties. Participants with incomplete data were excluded, leaving 49 (84.5%) participants in the analytic sample: 25 of 49 (51.0%) in the dementia care unit, 12 of 49 (24.5%) in the general care unit, and 12 of 49 (24.5%) in the short-stay unit (Figure 3). Participant numbers remained relatively stable across the ABAB phases, although a COVID-19 outbreak between March 2025 and April 2025 led to a temporary suspension of the intervention and missing data for that period.

Demographic characteristics are summarized in Table 2. Among the 49 analyzed participants, 40 (81.6%) were women and 9 (18.4%) were men. Age was collected in categories: 2 (4.1%) participants were aged 10 to 19 years, 4 (8.2%) were in their 20 to 29 years, 3 (6.1%) were in their 30 to 39 years, 16 (32.7%) were in their 40 to 49 years, 19 (38.8%) were in their 50 to 59 years, and 5 (10.2%) were aged 60 years or older. Most participants (n=41, 83.7%) were care workers, whereas 7 (14.3%) were nurses and 1 (2.0%) belonged to another profession. Regarding living status, 39 (79.6%) participants lived with family members, 7 (14.3%) lived alone, and 3 (6.1%) did not respond. Regarding disaster experience, 38 (77.6%) participants reported that their homes or families had been affected by the 2024 Noto Peninsula earthquake or the subsequent Oku-Noto torrential rains.

**Table 2.** Baseline demographic and occupational characteristics of the analytical sample by unit.

Characteristic	Dementia care unit (n=25), n (%)	General care unit (n=12), n (%)	Short-stay unit (n=12), n (%)	Total (N=49), n (%)
Gender				
Men	3 (12)	4 (33.3)	2 (16.7)	9 (18.4)
Women	22 (88)	8 (66.7)	10 (83.3)	40 (81.6)
Age (y)				
10-19	0 (0)	2 (16.7)	0 (0)	2 (4.1)
20-29	2 (8)	1 (8.3)	1 (8.3)	4 (8.2)
30-39	1 (4)	1 (8.3)	1 (8.3)	3 (6.1)
40-49	9 (36)	4 (33.3)	3 (25)	16 (32.7)
50-59	12 (48)	4 (33.3)	3 (25)	19 (38.8)
≥60	1 (4)	0 (0)	4 (33.3)	5 (10.2)
Profession				
Nurse	6 (24)	1 (8.3)	0 (0)	7 (14.3)
Care worker	19 (76)	10 (83.3)	12 (100)	41 (83.7)
Other	0 (0)	1 (8.3)	0 (0)	1 (2.0)
Living status				
Living with family	21 (84)	10 (83.3)	8 (66.7)	39 (79.6)
Living alone	2 (8)	2 (16.7)	3 (25)	7 (14.3)
No response	2 (8)	0 (0)	1 (8.3)	3 (6.1)
Disaster experience				
Yes	16 (64)	10 (83.3)	12 (100)	38 (77.6)
No	9 (36)	2 (16.7)	0 (0)	11 (22.4)

### Health-Related QoL and Well-Being

The median and IQR values for EQ-5D-5L utility, WHO-5, and MHC-SF total scores are presented in Table 3. Friedman tests detected no significant differences across the 5 study phases in any unit, and no post hoc comparisons were significant after Bonferroni correction. In the dementia care unit, no phase effect was observed for EQ-5D-5L (*P*=.08; Kendall *W*=0.076, negligible), WHO-5 (*P*=.36; Kendall *W*=0.030, negligible), or MHC-SF (*P*=.36; Kendall *W*=0.022, negligible). In the general care unit, no phase effect was observed for EQ-5D-5L (*P*=.74; Kendall *W*=0.044, negligible), WHO-5 (*P*=.20; Kendall *W*=0.137, negligible), or MHC-SF (*P*=.83; Kendall *W*=0.007, negligible). In the short-stay care unit, no phase effect was observed for EQ-5D-5L (*P*=.09; Kendall *W*=0.161, negligible), WHO-5 (*P*=.91; Kendall *W*=0.031, negligible), or MHC-SF (*P*=.75; Kendall *W*=0.084, negligible). In the pooled analysis, no phase effect was observed for EQ-5D-5L (*P*=.10; Kendall *W*=0.032, negligible), WHO-5 (*P*=.70; Kendall *W*=0.016, negligible), or MHC-SF (*P*=.44; Kendall *W*=0.022, negligible).

**Table 3.** Scores for health-related quality of life (QoL) and well-being measures according to unit and study phase.^a^

Measures and unit	Baseline, median (IQR)	A1, median (IQR)	B1, median (IQR)	A2, median (IQR)	B2, median (IQR)	*P* value	Kendall *W*	Effect size magnitude
EQ-5D-5L								
Dementia care unit	0.95 (0.95-1.00)	1.00 (0.95-1.00)	0.95 (0.90-1.00)	0.95 (0.90-1.00)	1.00 (0.95-1.00)	.08	0.076	Negligible
General care unit	0.95 (0.95-1.00)	1.00 (0.95-1.00)	1.00 (0.95-1.00)	1.00 (0.95-1.00)	1.00 (0.95-1.00)	.74	0.044	Negligible
Short-stay unit	0.90 (0.85-0.93)	0.95 (0.88-0.95)	0.90 (0.85-0.95)	0.95 (0.90-1.00)	0.95 (0.90-0.97)	.09	0.161	Small
Total	0.95 (0.90-1.00)	0.95 (0.93-1.00)	0.95 (0.90-1.00)	0.95 (0.90-1.00)	0.95 (0.95-1.00)	.10	0.032	Negligible
WHO-5^b^								
Dementia care unit	58 (48-68)	60 (48-71)	58 (48-76)	60 (48-76)	60 (53-72)	.36	0.030	Negligible
General care unit	68 (52-78)	72 (58-80)	60 (52-76)	60 (52-60)	60 (48-74)	.20	0.137	Small
Short-stay unit	60 (36-72)	60 (40-80)	60 (32-76)	56 (38-78)	52 (38-80)	.91	0.031	Negligible
Total	60 (47-76)	60 (48-80)	60 (48-76)	60 (48-76)	60 (48-76)	.70	0.016	Negligible
MHC-SF^c^								
Dementia care unit	42 (28-50)	38 (26-48)	39 (29-43)	41 (28-49)	37 (25-45)	.36	0.022	Negligible
General care unit	36 (15-48)	36 (15-45)	41 (28-48)	41 (25-53)	42 (25-43)	.83	0.007	Negligible
Short-stay unit	30 (24-42)	28 (24-38)	38 (18-41)	34 (20-40)	30 (18-41)	.75	0.084	Negligible
Total	39 (26-48)	36 (23-45)	39 (26-43)	39 (27-45)	37 (23-42)	.44	0.022	Negligible

a Median (IQR) with *P* values from Friedman tests for repeated measures are reported.

b WHO‑5: World Health Organization–5 Well‑Being Index.

c MHC‑SF: Mental Health Continuum–Short Form.

### Safety Outcomes

Table 4 presents the results for perceived safety. For the items “collisions with the robot,” “falls or accidents caused by the robot,” and “feeling unsafe when the robot was present,” no significant differences were observed between the 2 intervention phases in any unit or in the pooled analysis. Rank-biserial correlations ranged from negligible to large in magnitude across units, but all adjusted *P* values were nonsignificant. No adverse events caused by the robot were reported.

**Table 4.** Safety outcomes between A1 and A2 across units.^a^

Unit and items	A1, median (IQR)	A2, median (IQR)	Median (IQR) difference	*P* value (adjusted *P* value)	Rank-biserial correlation	Effect size magnitude
Dementia care unit						
Felt unsafe	1.5 (1-3)	1.5 (1-3)	0 (−0.75 to 1)	.95 (>.99)	0.018	Negligible
Falls or accidents caused by the robot	1 (1-1.75)	1 (1-1)	0 (0 to 0)	.27 (>.99)	−0.382	Medium
Collisions with the robot	1 (1-3)	1 (1-2.75)	0 (−1.75 to 0)	.47 (>.99)	−0.231	Small
General care unit						
Felt unsafe	1 (1-1)	2 (1-2.25)	0 (0 to 1)	.20 (>.99)	0.571	Large
Falls or accidents caused by the robot	1 (1-1)	1 (1-1)	0 (0 to 0)	.41 (>.99)	0.500	Large
Collisions with the robot	1 (1-2.25)	1 (1-2.25)	0 (0 to 0.25)	.89 (>.99)	0.067	Negligible
Short-stay unit						
Felt unsafe	2 (1-2)	2 (1-2)	0 (−1 to 1)	>.99 (>.99)	0.000	Negligible
Falls or accidents caused by the robot	1 (1-1.25)	1 (1-3)	0 (0 to 2)	.09 (>.99)	0.714	Large
Collisions with the robot	3 (1-4)	3 (2.75-3.25)	0 (−0.25 to 1.25)	.56 (>.99)	0.222	Small
Total units						
Felt unsafe	1 (1-2)	2 (1-2.75)	0 (−0.75 to 1)	.51 (>.99)	0.130	Small
Falls or accidents caused by the robot	1 (1-1)	1 (1-1)	0 (0 to 0)	.50 (>.99)	0.129	Small
Collisions with the robot	1 (1-3)	1.5 (1-3)	0 (−0.75 to 0)	.61 (>.99)	−0.068	Negligible

a Median (IQR) scores, along with median differences between A1 and A2, unadjusted and Bonferroni-adjusted *P* values from the Wilcoxon signed rank tests, are reported.

### Acceptability Outcomes

Table 5 displays the results of perceived acceptability. In the dementia care unit, the semantic differential items “friendly” (*P*=.01; rank-biserial correlation=0.516; large), “made me feel calm” (*P*=.045; *r*=0.571; large), “like” (*P*=.03; *r*=0.559; large), and “felt at peace” (*P*=.02; *r*=0.718; large) showed nominal unadjusted increases. Interaction frequency items also showed nominal increases, with large effect sizes for stroking (*r*=0.733), touching (*r*=0.724), interacting (*r*=0.848), and holding (*r*=0.737). However, none remained significant after Bonferroni correction (adjusted *P*=.32, .89, .51, .39, .43, .12, .11, respectively).

**Table 5.** Acceptability outcomes between A1 and A2 across units.^a^

Unit and items	A1, median (IQR)	A2, median (IQR)	Median difference (IQR difference)	*P* value (adjusted *P* value)	Rank-biserial correlation	Effect size magnitude
Dementia care unit						
Not cute or cute	5 (4-5)	5 (4-5)	0 (0 to 0)	>.99 (>.99)	0.000	Negligible
Unfriendly or friendly	3 (3-4)	4 (4-4.75)	0 (0 to 1)	.01 (.32)	0.516	Large
Unkind or kind	4 (3-4.75)	4 (3-4)	0 (0 to 0.75)	.48 (>.99)	0.227	Small
Felt a cold impression or felt warm and fuzzy	4 (4-5)	4 (4-5)	0 (0 to 0.75)	.66 (>.99)	0.132	Small
Made me feel agitated or made me feel calm	3 (3-4)	4 (3.25-4)	0 (0 to 1)	.045 (>.99)	0.571	Large
Would not like to be friends with it or would like to be friends with it	4 (3-4)	4 (3.25-4.75)	0 (0 to 1)	.50 (>.99)	0.198	Small
Dislike or like	4 (3-4.5)	4 (4-5)	0 (0 to 1)	.03 (.89)	0.559	Large
Would not like to use it at home or would like to use it at home	3 (2-4)	3 (3-3.5)	0 (−0.5 to 1)	.71 (>.99)	0.098	Negligible
Thoughtless or thoughtful	3 (3-4)	3 (3-4)	0 (0 to 1)	.24 (>.99)	0.352	Medium
Incompetent or competent	4 (3-4)	4 (3-4)	0 (0 to 1)	.06 (>.99)	0.590	Large
Did not feel soothed or felt soothed	4 (4-5)	4 (4-5)	0 (0 to 0)	.61 (>.99)	−0.154	Small
Unintelligent or intelligent	3 (3-4)	4 (3-4)	0 (0 to 1)	.06 (>.99)	0.590	Large
No knowledge or knowledge	3 (3-4)	4 (3-4)	0 (0 to 1)	.10 (>.99)	0.564	Large
Bad impression or good impression	4 (3.5-5)	4 (3-4)	0 (0 to 1)	.41 (>.99)	0.238	Small
Unpleasant atmosphere or pleasant atmosphere	4 (3.5-5)	4 (4-5)	0 (0 to 1)	.27 (>.99)	0.314	Medium
Unapproachable or approachable	4 (3-5)	4 (4-5)	0 (0 to 1)	.08 (>.99)	0.736	Large
Felt surprised or felt at peace	3 (3-4)	4 (4-4)	0 (0 to 1)	.02 (.51)	0.718	Large
How often did you stroke the robot?	2 (1-3)	3 (2-4)	0 (0 to 2)	.02 (.39)	0.733	Large
How often did you touch the robot?	2 (1-3)	3 (2-4)	0 (0 to 2)	.02 (.43)	0.724	Large
How often did you interact with the robot?	2 (1-3)	3 (2.25-4)	0 (0 to 1.75)	.004 (.12)	0.848	Large
How often did you hold the robot?	1 (0-2)	2 (1.25-3)	1 (0 to 2)	.004 (.11)	0.737	Large
General care unit						
Not cute or cute	5 (3.75-5)	4 (3-5)	0 (−1 to 0)	.06 (>.99)	−1.000	Large
Unfriendly or friendly	3.5 (3-4.25)	3 (3-4)	0 (−0.25 to 0)	.48 (>.99)	−0.762	Large
Unkind or kind	3 (3-5)	3 (3-4.25)	0 (0 to 0)	.65 (>.99)	−0.333	Medium
Felt a cold impression or felt warm and fuzzy	4.5 (3.75-5)	3 (3-4)	–0.5 (−1.25 to 0)	.046 (>.99)	−0.821	Large
Made me feel agitated or made me feel calm	3.5 (3-4)	3 (3-4)	0 (0 to 0)	.71 (>.99)	−0.200	Small
Would not like to be friends with it or would like to be friends with it	3.5 (3-5)	3 (3-4)	0 (−0.25 to 0)	.10 (>.99)	−1.000	Large
Dislike or like	4 (3-5)	3 (3-4)	–0.5 (−1 to 0)	.02 (.62)	−1.000	Large
Would not like to use it at home or would like to use it at home	3 (2.75-3.25)	3 (2-4)	0 (−0.25 to 0)	.74 (>.99)	0.143	Small
Thoughtless or thoughtful	3 (3-3.25)	3 (3-3.25)	0 (0 to 0)	.56 (>.99)	0.333	Medium
Incompetent or competent	3 (3-4)	3 (3-4)	0 (0 to 1)	.32 (>.99)	0.429	Medium
Did not feel soothed or felt soothed	4 (3-5)	3.5 (3-4.25)	0 (−0.25 to 0)	.33 (>.99)	−0.467	Medium
Unintelligent or intelligent	3 (3-4)	3 (3-3.25)	0 (0 to 0.25)	.48 (>.99)	0.333	Medium
No knowledge or knowledge	3 (3-4)	3 (3-4)	0 (0 to 0.25)	.26 (>.99)	0.600	Large
Bad impression or good impression	3.5 (3-5)	3.5 (3-4.25)	0 (−0.25 to 0)	.48 (>.99)	−0.333	Medium
Unpleasant atmosphere or pleasant atmosphere	3.5 (3-5)	3 (3-4.25)	0 (−0.25 to 0)	.26 (>.99)	−0.600	Large
Unapproachable or approachable	4.5 (3-5)	3.5 (3-4)	0 (−1 to 0)	.08 (>.99)	−0.333	Medium
Felt surprised or felt at peace	3 (3-4)	3 (3-4)	0 (0 to 0.25)	.71 (>.99)	0.200	Small
How often did you stroke the robot?	3 (2.5-4)	1 (0-2.25)	–2 (−3 to 0)	.02 (.61)	−0.844	Large
How often did you touch the robot?	3 (2.5-4)	1 (0-2.25)	–2 (−2.25 to 0)	.02 (.48)	−0.836	Large
How often did you interact with the robot?	3 (3-4)	1 (0.75-2.25)	–2 (−2.25 to 0)	.007 (.19)	−1.000	Large
How often did you hold the robot?	3 (0.75-3)	0.5 (0-1.25)	–0.5 (−2.25 to 0)	.03 (.88)	−0.893	Large
Short-stay units						
Not cute or cute	5 (4-5)	5 (4-5)	0 (0 to 0.25)	.32 (>.99)	0.500	Large
Unfriendly or friendly	4 (3-5)	4 (3-52)	0 (−0.25 to 0)	.48 (>.99)	−0.500	Large
Unkind or kind	4 (4-5)	4 (4-5)	0 (−1 to 0)	.10 (>.99)	−0.714	Large
Felt a cold impression or felt warm and fuzzy	5 (4-5)	4 (3-4)	0 (−1 to 0)	.53 (>.99)	−0.250	Small
Made me feel agitated or made me feel calm	4 (3-5)	4 (3-5)	0 (0 to 0)	.41 (>.99)	−0.500	Large
Would not like to be friends with it or would like to be friends with it	4.5 (4-5	4 (3.75-4.25)	0 (−1 to 0)	.10 (>.99)	−0.714	Large
Dislike or like	5 (4-5)	4 (3.75-5)	0 (−1 to 0)	.21 (>.99)	−0.500	Large
Would not like to use it at home or would like to use it at home	4 (3.75-5)	3.5 (3-4)	–0.5 (−1 to 0)	.31 (>.99)	−0.389	Medium
Thoughtless or thoughtful	4 (3.75-5)	3.5 (3-4.25)	0 (−1 to 0.25)	.56 (>.99)	−0.222	Small
Incompetent or competent	5 (4.75-5)	4 (4-5)	0 (−1 to 0)	.21 (>.99)	−0.500	Large
Did not feel soothed or felt soothed	5 (3-5)	4.5 (3-5)	0 (0 to 0)	>.99 (>.99)	0.000	Negligible
Unintelligent or intelligent	4 (4-5)	4.5 (3-5)	0 (−1 to 0.25)	.53 (>.99)	−0.250	Small
No knowledge or knowledge	5 (4-5)	4.5 (3.75-5)	0 (−0.25 to 0.25)	.74 (>.99	−0.143	Small
Bad impression or good impression	5 (4-5)	5 (4-5)	0 (0 to 0)	.71 (>.99)	−0.200	Small
Unpleasant atmosphere or pleasant atmosphere	5 (4-5)	4 (4-5)	0 (−1 to 0)	.16 (>.99)	−0.667	Large
Unapproachable or approachable	5 (4-5)	4 (4-5)	0 (−1 to 0)	.21 (>.99)	−0.333	Medium
Felt surprised or felt at peace	5 (3-5)	4 (3-5)	0 (−0.25 to 0)	.48 (>.99)	−0.333	Medium
How often did you stroke the robot?	4 (3-4)	4 (2.75-4)	0 (0 to 0)	.71 (>.99)	−0.200	Small
How often did you touch the robot?	4 (3-4)	3.5 (3-4)	0 (−0.25 to 0)	.66 (>.99)	−0.200	Small
How often did you interact with the robot?	4 (3-4)	3 (3-4)	0 (−1 to 0)	.41 (>.99)	−0.333	Medium
How often did you hold the robot?	3 (2.75-4)	3 (2.75-4)	0 (0 to 0)	>.99 (>.99)	0.000	Negligible
Total units						
Not cute or cute	5 (4-5)	4 (4-5)	0 (0 to 0)	.25 (>.99)	−0.205	Small
Unfriendly or friendly	3 (3-4)	4 (3-4)	0 (0 to 1)	.09 (>.99)	−0.060	Negligible
Unkind or kind	3 (3-5)	4 (3-4)	0 (0 to 0)	.71 (>.99)	−0.142	Small
Felt a cold impression or felt warm and fuzzy	4 (4-5)	4 (3-4.75)	0 (0 to 1)	.33 (>.99)	−0.230	Small
Made me feel agitated or made me feel calm	3 (3-4)	4 (3-4)	0 (0 to 1)	.15 (>.99)	0.210	Small
Would not like to be friends with it or would like to be friends with it	4 (3-5)	4 (3-4)	0 (−0.75 to 0)	.83 (>.99)	−0.237	Small
Dislike or like	4 (3-5)	4 (3-5)	0 (−1 to 1)	.74 (>.99)	−0.062	Negligible
Would not like to use it at home or would like to use it at home	3 (3-5)	3 (3-4)	0 (−1 to 1)	.59 (>.99)	−0.008	Negligible
Thoughtless or thoughtful	3 (3-4)	3 (3-4)	0 (0 to 1)	.19 (>.99)	0.153	Small
Incompetent or competent	3 (3-4)	4 (3-4)	0 (0 to 1)	.03 (.86)	0.255	Small
Did not feel soothed or felt soothed	4 (3-5)	4 (3-5)	0 (0 to 0)	.31 (>.99)	−0.219	Small
Unintelligent or intelligent	3 (3-4)	3 (3-4)	0 (0 to 1)	.045 (>.99)	0.297	Small
No knowledge or knowledge	3 (3-4)	4 (3-4)	0 (0 to 1)	.047 (>.99)	0.362	Medium
Bad impression or good impression	4 (3-5)	4 (4-5)	0 (0 to 0.5)	.72 (>.99)	0.033	Negligible
Unpleasant atmosphere or pleasant atmosphere	4 (3-5)	4 (3.5-5)	0 (0 to 0)	.68 (>.99)	−0.062	Negligible
Unapproachable or approachable	4 (3-5)	4 (3.5-5)	0 (0 to 0.5)	.72 (>.99)	0.254	Small
Felt surprised or felt at peace	3 (3-4)	4 (3-4)	0 (0 to 1)	.03 (.79)	0.359	Medium
How often did you stroke the robot?	3 (1-4)	3 (1-4)	0 (−1 to 1)	.78 (>.99)	0.042	Negligible
How often did you touch the robot?	3 (2-4)	3 (2-4)	0 (−1 to 1)	.91 (>.99)	0.021	Negligible
How often did you interact with the robot?	3 (2-3.75)	3 (1-4)	0 (−1 to 1)	.81 (>.99)	−0.071	Negligible
How often did you hold the robot?	1 (0-3)	2 (0-3)	0 (−0.75 to 2)	.32 (>.99)	0.204	Small

a Median (IQR) scores, along with median differences between A1 and A2, unadjusted and Bonferroni-adjusted *P* values from the Wilcoxon signed rank tests, are reported.

In the general care unit, nominal unadjusted decreases were observed for “felt warm and fuzzy” (*P*=.046; *r*=−0.821; large) and “like” (*P*=.02; *r*=−1.000; large), along with lower interaction frequency ratings. However, none remained significant after Bonferroni correction (adjusted *P*>.99 and *P=*.63, respectively).

In the pooled analysis, the items “competent” (*P*=.03; *r*=0.255; small), “intelligent” (*P*=.45; *r*=0.297; small), “knowledgeable” (*P*=.047; *r*=0.362; medium), and “felt at peace” (*P*=.03; *r*=0.359; medium) showed nominal unadjusted differences, but none remained statistically significant after Bonferroni correction (adjusted *P*>.99, *P*>.99, and *P=*.79, respectively).

Median scores and interquartile ranges, along with median differences between A1 and A2, unadjusted and Bonferroni-adjusted *P* values from Wilcoxon signed rank tests.

### Intention to Continue Use

No intention to continue use item remained statistically significant after Bonferroni correction in any unit (Table 6). In the short-stay unit, “unnecessary in my facility” showed a nominal unadjusted decrease (*P*=.03; rank-biserial correlation=−1.000, large), but this was not significant after adjustment (adjusted *P*=.68).

**Table 6.** Intention to continue use outcomes between A1 and A2 across units.^a^

Unit and items	A1, median (IQR)	A2, median (IQR)	Median difference (IQR difference)	*P* value (adjusted *P* value)	Rank-biserial correlation	Effect size magnitude
Dementia care unit						
No interest in LOVOT	3 (1-3)	2 (1-3)	0 (−0.75 to 1)	.95 (>.99)	−0.017	Negligible
Bored with LOVOT	2 (1-3)	2 (1-3)	0 (0 to 0)	.48 (>.99)	−0.231	Small
Unnecessary in my facility	3 (2-3)	2 (1-3)	0 (−1 to 0)	.39 (>.99)	−0.257	Small
General care unit						
No interest in LOVOT	3 (2-3)	3 (3-3)	0 (0 to 1)	.67 (>.99)	0.179	Small
Bored with LOVOT	3 (1.75-3)	3 (3-3)	0 (0 to 1)	.33 (>.99)	0.467	Medium
Unnecessary in my facility	2.5 (2-3)	3 (2.75-3)	0 (0 to 0.25)	.26 (>.99)	0.600	Large
Short-stay unit						
No interest in LOVOT	3 (2-4)	2 (2-3)	0 (−1.25 to 0)	.10 (>.99)	−0.800	Large
Bored with LOVOT	2 (2-2.25)	2 (2-2.25)	0 (0 to 0)	>.99 (>.99)	0.000	Negligible
Unnecessary in my facility	3 (2.75-3)	2 (2-3)	0 (−1 to 0)	.03 (>.68)	−1.000	Large
Total units						
No interest in LOVOT	3 (1-3)	3 (1-3)	0 (0 to 1)	.78 (>.99)	−0.114	Small
Bored with LOVOT	3 (1-3)	2 (1-3)	0 (0 to 0.75)	.96 (>.99)	−0.005	Negligible
Unnecessary in my facility	3 (2-3)	3 (1.25-3)	0 (−1 to 0)	.69 (>.99)	−0.246	Small

a Median (IQR) scores, along with median differences between A1 and A2, unadjusted and Bonferroni-adjusted *P* values from the Wilcoxon signed rank tests, are reported.

## Discussion

### Principal Findings and Novelty of This Study

In this exploratory pilot study, the short-term deployment of a nonverbal AI communication robot (LOVOT) was not associated with significant changes in health-related QoL or well-being among staff working in disaster-affected care facilities following the 2024 Noto Peninsula earthquake and the subsequent Oku-Noto torrential rains. We also evaluated safety, acceptability, and intention to continue use, and no robot-related adverse events were reported. Although several nominal unadjusted differences were observed in some acceptability and interaction frequency items across individual units and in the pooled analysis, none remained statistically significant after Bonferroni correction. These findings, therefore, should be interpreted as exploratory and hypothesis-generating rather than confirmatory.

Previous studies have examined nonverbal AI communication robots primarily in older adults; however, to the best of our knowledge, no study has focused on care staff in institutional settings [5,11,12]. Notably, 38 of 49 (78%) participants reported that their homes or families had been affected by the 2024 disasters, placing this intervention in an unusual postdisaster context. To date, no study has simultaneously examined QoL, well-being, safety, acceptability, and intention to continue use of a nonverbal AI communication robot among care staff working under disaster conditions.

### Effects on Health-Related QoL and Well-Being

The pooled effect sizes for the primary outcomes were negligible (Kendall *W*=0.032 for EQ-5D-5L, 0.016 for WHO-5, and 0.022 for MHC-SF), suggesting that any overall phase-related changes were very small, even beyond statistical nonsignificance. Previous studies have reported an average WHO-5 score of 64.2 points in the general population [19]. The median WHO-5 score of 60 points in our participants was slightly lower, suggesting that disaster-related stress may negatively affect mental health and require psychological support. Although some interaction frequency items showed nominal unadjusted increases, particularly in the dementia care unit, these differences did not remain significant after correction for multiple comparisons. QoL encompasses physical, psychological, and social domains [25], and our intervention period of 2×2 weeks may have been significantly short to influence the overall QoL. Furthermore, EQ-5D-5L scores at baseline were comparable to those of healthy Japanese adults, possibly owing to the fact that more than 1 year had elapsed since the disasters [17], making it difficult to detect additional improvements. Similarly, the intervention did not significantly improve well-being. Given the low baseline WHO-5 scores, one might expect a beneficial effect; however, prior work has suggested that nonverbal AI robots can produce only transient improvements in mood and social engagement among people with dementia [13]. The WHO-5 reflects psychological health and positive affect over a 2-week period, and such constructs may change more slowly than physical or behavioral outcomes [26]. Thus, longer intervention durations and follow-up assessments may be required to observe meaningful changes.

### Safety, Acceptability, and Continued Use

In the pooled analysis, no statistically significant differences were observed between the 2 intervention periods (A1 and A2) in safety or intention to continue use. None of the units reported accidents or incidents attributable to the robot. These findings support the short-term feasibility of deploying the robot in staff work environments under disaster-affected conditions. Although the robot is equipped with sensors to avoid obstacles, it cannot perfectly avoid all objects; therefore, future implementation studies should refine placement and operational protocols, particularly in areas where frail older adults may enter staff spaces.

At the same time, the lack of statistically significant findings after Bonferroni correction does not necessarily preclude potentially meaningful experiential signals. In the pooled analysis, several acceptability items showed nominal unadjusted differences in a favorable direction, including “competent” (*P*=.03; *r*=0.255), “intelligent” (*P*=.045; *r*=0.297), “knowledgeable” (*P*=.047; *r*=0.362), and “felt at peace” (*P*=.03; *r*=0.359). Although none of these findings remained statistically significant after adjustment, their consistent direction and small-to-medium effect sizes suggest that repeated exposure may have modestly strengthened perceptions of the robot as reassuring and socially intelligible. These pooled findings should therefore be interpreted as exploratory.

This pattern was more pronounced in the dementia care unit, where several nominal unadjusted acceptability changes were accompanied by large effect sizes, including “friendly” (*P*=.02; *r*=0.516), “made me feel calm” (*P*=.045; *r*=0.571), “like” (*P*=.03; *r*=0.559), and “felt at peace” (*P*=.02; *r*=0.718). Interaction frequency items also increased, with large effect sizes for stroking (*P*=.02; *r*=0.733), touching (*P*=.02; *r*=0.724), interacting with the robot (*P*=.008; *r*=0.848), and holding the robot (*P*=.004; *r*=0.737). Although these findings did not remain statistically significant after Bonferroni correction, the convergence of affective and behavioral changes may indicate growing familiarity and attachment under repeated exposure. This possibility warrants examination in adequately powered studies.

By contrast, the general care unit showed nominal unadjusted decreases in some acceptability and interaction frequency items, several with large negative effect sizes, such as “felt warm and fuzzy” (*P*=.046; *r*=−0.821), “like” (*P*=.02; *r*=−1.000), stroking (*P*=.02; *r*=−0.844), touching (*P*=.02; *r*=−0.836), interacting with the robot (*P*=.007; *r*=−1.000), and holding the robot (*P*=.03; *r*=−0.893). These patterns emerged after the COVID-19 outbreak that occurred between A1 and A2 and may reflect altered workload, infection control demands, and reduced willingness to physically engage with shared objects rather than a simple decline in robot acceptability.

In the short-stay unit, the item “The robot is unnecessary in my facility” showed a nominal unadjusted decrease (*P*=.03; *r*=−1.000), suggesting a possible increase in perceived usefulness. Because the short-stay unit serves more ambulatory clients than the dementia and general care units, staff may have observed more independent interactions between residents and the robot, which may have contributed to this tendency. However, this finding was also exploratory and did not remain statistically significant after adjustment.

### Limitations

This study has some limitations. First, we did not conduct a formal sample size calculation because this was a pragmatic, exploratory pilot study conducted under postdisaster service constraints, and all eligible staff in the participating units were recruited. Therefore, the study was not powered for definitive effectiveness testing, and the null findings should not be interpreted as evidence of no effect.

Second, because the robot was visibly present in the workplace, neither the participants nor the researchers could be blinded to the intervention. Awareness of the intervention and study participation may have influenced staff behavior (ie, Hawthorne effect).

Third, although contamination was reduced by staggered unit start dates, physical separation between units, instructions to staff not to use the robot outside the assigned unit, the use of a different facility for the short-stay unit, cross-unit contamination, and other contextual influences cannot be fully excluded.

Fourth, the ABAB sequence was fixed rather than randomized. Therefore, order effects, period effects, habituation effects, and carryover effects cannot be separated from intervention effects.

Fifth, a COVID-19 outbreak between March 2025 and April 2025 led to the temporary suspension of the intervention, missing data, increased workload, and stricter infection control procedures. These factors may have independently affected staff well-being and willingness to physically interact with the robot, thereby influencing the primary outcomes and acceptability ratings. This is particularly important when interpreting differences between A1 and A2, because the contextual conditions were not equivalent across the 2 intervention periods.

Sixth, although robot use was confirmed through a dedicated application, we did not have validated participant-level objective exposure data. Because the robot was shared within each unit, the logs could not be attributed reliably to specific staff members. Therefore, interaction was assessed pragmatically using phase-end questionnaire items rather than objective individual-level measures.

Finally, the same robot type was used for all participants, irrespective of individual preferences, and the frequency, duration, and timing of interactions were not standardized. These factors may have introduced heterogeneity in exposure and response. Accordingly, the unit-level acceptability patterns observed in this study should be interpreted cautiously and regarded as preliminary signals for future investigation rather than as evidence of differential effectiveness.

### Conclusions

Among staff working in disaster-affected care facilities, the introduction of a nonverbal AI communication robot did not significantly improve QoL or well-being. Short-term deployment appeared feasible and was not associated with reported adverse events, but its efficacy as a mental health support intervention remains unproven. Although several acceptability and interaction-related items showed favorable exploratory signals, particularly in the dementia care unit, these findings did not remain statistically significant after correction for multiple comparisons. The exploratory acceptability findings may help generate hypotheses for future adequately powered studies.

## Supplementary material

10.2196/89166 Multimedia Appendix 1 Assessment instruments used to evaluate health-related quality of life, well-being, safety, acceptability, interaction frequency, and intention to continue use.

## Abbreviations

AI: artificial intelligence
MHC‑SF: Mental Health Continuum–Short Form
QoL: quality of life
WHO‑5: World Health Organization–5 Well‑Being Index
