# Supplementary material for: Nonverbal AI-Based Communication Robot for Staff in Disaster-Affected Care Facilities: Exploratory ABAB Intervention Study
Source: JMIR Form Res. 2026 Jul 14;10:e89166. doi: 10.2196/89166 (PMC13367946; doi:10.2196/89166)
Supplement: Multimedia Appendix 1 [file formative-v10-e89166-s001.docx]

Multimedia Appendix 1: Assessment Instruments

**EQ‑5D‑5L**

This generic preference‑based measure of health status comprises five dimensions—mobility, self‑care, usual activities, pain/discomfort, and anxiety/depression—each rated on five levels. Responses were converted to utility values using the Japanese value set; higher values indicate better health-related QoL. The EQ‑5D‑5L has demonstrated satisfactory validity and reliability across diverse populations.

**WHO‑5 Well‑Being Index**

The WHO‑5 evaluates positive subjective well‑being over the past two weeks using five items scored from 0 (“At no time”) to 5 (“All of the time”). The raw sum (0–25) is multiplied by four to obtain a percentage score (0–100 %); scores below 50 % indicate poor well‑being. The Japanese version has shown good psychometric properties.

**Mental Health Continuum–Short Form (MHC‑SF)**

The MHC‑SF includes 14 items covering emotional, social, and psychological well‑being, each rated from 0 (“Never”) to 5 (“Every day”). Total scores range from 0 to 70, with higher scores reflecting greater well‑being. The instrument has been validated in Japanese and international samples.

**Safety assessment**

Safety was assessed using **three negatively worded statements** rated on a five-point Likert scale:
1 = strongly disagree, 2 = disagree, 3 = neutral, 4 = agree, 5 = strongly agree.

**Items:**

1. “I felt unsafe when the robot was present.”
2. “There were falls or accidents caused by the robot.”
3. “There were collisions with the robot (e.g., kicking, tripping).”

**Higher scores indicate *greater perceived risk* (i.e., worse safety).**
This directionality matches the statistical analysis reported in the manuscript.
Actual physical incidents were also recorded.

**Acceptability assessment**

Acceptability was assessed using 17 bipolar adjective pairs rated on a five-point semantic differential scale (1 = negative adjective, 5 = positive adjective). The items were as follows:

1. not cute / cute

2. unfriendly / friendly

3. unkind / kind

4. felt a cold impression / felt warm and fuzzy

5. made me feel agitated / made me feel calm

6. would not like to be friends with it / would like to be friends with it

7. dislike / like

8. would not like to use it at home / would like to use it at home

9. thoughtless / thoughtful

10. incompetent / competent

11. did not feel soothed / felt soothed

12. unintelligent / intelligent

13. no knowledge / knowledgeable

14. bad impression / good impression

15. unpleasant atmosphere / pleasant atmosphere

16. unapproachable / approachable

17. felt surprised / felt at peace

Higher scores indicate more favorable acceptability.

These adjective pairs correspond to the acceptability constructs analyzed in the manuscript (e.g., calm and felt at peace).

**Interaction frequency**

Interaction (petting, touching, engaging with, or holding the robot) was rated on a six-point scale:
0 = never, 1 = once or twice, 2 = twice per fortnight, 3 = 4–6 times per fortnight, 4 = almost daily, 5 = daily.

**Intention to continue use**

The following three negatively worded statements were rated on a five-point Likert scale (1 = strongly disagree, 5 = strongly agree):

1. “I have no interest in LOVOT.”
2. “I feel bored with LOVOT.”
3. “I think LOVOT is unnecessary in my facility.”

**Higher scores indicate lower willingness to continue use.**
This scoring direction matches the interpretation used in the statistical analyses.
